# Home range variation and site fidelity of Bornean southern gibbons [*Hylobates albibarbis*] from 2010-2018

**DOI:** 10.1371/journal.pone.0217784

**Published:** 2019-07-31

**Authors:** Susan M. Cheyne, Bernat Ripoll Capilla, Abdulaziz K., Supiansyah, Adul, Eka Cahyaningrum, David Ehlers Smith

**Affiliations:** 1 Borneo Nature Foundation, Palangka Raya, Central Kalimantan, Indonesia; 2 Faculty of Social Sciences, Oxford Brookes University, Oxford, United Kingdom; 3 University of Kwa-Zulu Natal, Pietermaritzburg, South Africa; Chinese Academy of Forestry, CHINA

## Abstract

Gibbons are highly territorial and have two key areas within these territories. The core area in which we find all sleeping trees and the trees from which the gibbons duet and the wider home range (HR) which has varying levels of overlap with neighbouring gibbon groups. The core area is strenously defended, with the wider HR being more of a shared area for neighbouring groups. We present ranging and movement data on four wild gibbon groups from January 2010 to July 2018. Global Positioning System (GPS) data were collected every 5 mins on habitauted groups in Sebangau, Central Kalimantan, Indonesia resulting in 35,521 waypoints. Gibbon home- and corerange sizes were calculated using 95%, and 50%, volume contours of kernel density estimates. Home-ranges ranged from 58.74–147.75 ha with a mean of 95.7 ± SD 37.75 ha, the highest of comparable *Hylobates* species. Core-range size ranged from 20.7–51.31 ha with a mean size of 31.7 ± SD 13.76 ha. Gibbons had consistant site fidelity for their home- and core ranges; percentage overlap ranged from 4.3 23.97% with a mean 16.5 ± SD 8.65% overlap in home-range area. Core ranges did not overlap with the exception of two groups, in which a 0.64 ha (2.69%) overlap occurred. Unsurprisingly forest loss from fire does affect the location of the HR of the impacted group, but does not appear to affect adjacent groups, though more data are needed on this. Understanding the complex use of space of these territorial animals is important in assessing both carrying capacity for wild populations and understading how reintroduced gibbon pairs will establish their core and HR.

## Introduction

Home range is defined as the area in which an animal normally travels during routine activities, such as food gathering, mating and caring for young [1]. Home range estimation is important for the understanding of the species’ spatial and behavioural ecology [2–4]. Information about ranging patterns and its’ determinants are important for several reasons: this constitutes basic data for the study of social organization of a species, gives an indicator of the spatial needs for individuals and populations, and provides essential tools for conservation management, especially for species found in small, isolated or endangered habitats [2,5,6].

The first descriptions of territorial and space use in gibbons are given [7,8] and [9,10], studying the concept of territoriality, disputes between groups, and home range determinants. Global Positioning Systems (GPS) data allow researchers to analyse animal’s spatial use and relate it to behavioural and socio-ecology. It is useful to analyse the determinants which influence home range size and what influences home range sizes between groups in the same habitat [11–14]. Patterns of ranging behaviour can be influenced by the distribution and abundance of food trees [15–19], phenology [20,21], body size [22–24], group size [25–28], location of night trees [29–35], interaction between conspecific groups [36] and the need to patrol territorial boundaries [37–41]. Calculating home range overlap provides information about the area shared between groups, this area represents a shared space where gibbons compete for food resources. Territorial disputes between gibbons take place along the boundary where ranges overlap, and seems that these disputes appear to occur as a result of chance encounters between groups near the boundary [41]. Gibbon territories can be influenced by food availability, canopy cover [18,42,43], presence of tall emergent trees from which to sing [44–46] and suitable sleeping sites [30,33,34,47]. Spacing of gibbons is regulated by both direct encounters [9,41,48,49] and by singing [45] with elements of the song (duet or coda) travelling over varying distances thus carrying information both intra- and inter-group [46].

The study area was impacted by the forest fires in 2015, which caused widespread habitat loss across Borneo and Sumatra [50–53]. For this reason, we also want to understand how loss of forest could impact established home ranges for gibbons.

In this study we present the first long-term assessment on gibbon movement in a peat-swamp forest, specifically focussing on three key analyses:

1. Home range size and changes over time,
2. Home range location, site fidelity and changes over time,
3. Home range overlap between groups and changes over time and
4. Investigating the impact of the 2015 fires on gibbon home ranges.

## Materials and methods

**Fig 1:**
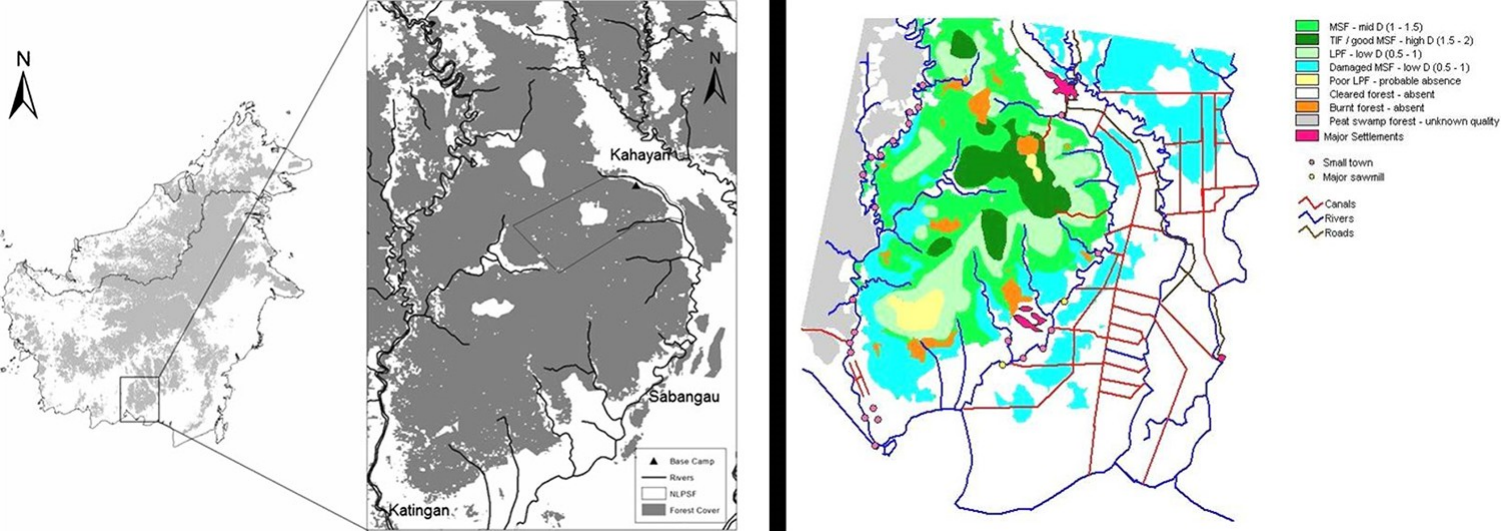
(a) Location of the Natural Laboratory for the Study of Peat-Swamp Forest (NLPSF) within Sebangau tropical peat-swamp forest and Borneo. Forest cover is shaded gray, non-forested areas white. Adapted from [5]. Fig 1b shows habitat breakdown of the landscape.

The study site is the National Laboratory of Peat Swamp Forest (NLPSF) managed by CIMTROP (Centre for the International Cooperation in management of Tropical Peatland). NLPSF is located at the NE part of the Sebangau Forest, Central Kalimantan, Indonesia (Fig 1). Sebangau catchment covers an area of 5600 km^2^ of peat-swamp forest [54,55]. The research area is 4 km^2^ of grid transects system, containing seven habituated gibbon groups. The Sebangau catchment is characterised by peat-swamp forest and low elevation, presenting three different forest types: mixed swamp forest, low pole forest and tall interior forest [56]. Our study was carried in Mixed Swamp Forest (MSF) which occupies 40% of the total area of Sebangau forest [57]. The MSF extends ~4km from the margin of the forest into the interior. It is beyond the locaiton of the river flooding zone. The forest is tall and stratified with an upper canopy at ~35m, a closed layer between 15-25m and an understorey of smaller trees at 7-12m. Trees grown on hummocks interspersed with hollows which fill with water during the wet season. Many of the species have stilt or butress root systems and pneumatophores are common. Typical trees of the upper and mid-canopy are *Aglaia rubinigosa*, *Calophyllum hosei*, *C*. *lowii*, *C*. *sclerophylum*, *Combretocarpus rotundatus*, *Cratoxylum galucum*, *Dactylocladus stenostachys*, *Dipterocarpus corieus*, *Dyera costulata*, *Ganua mottleyana*, *Gonstylua bancanus*, *Mezettia leptopoda* [58], *Neoscortechinia kingii*, *Palaquium cochlearifolium*, *P*. *leiocarpum*, *Shorea blangeran*, *S*. *teysmanniana* and *Xylopia fusca* [54,59].

GPS data collection began in May 2005 on seven groups but because of issues with historic satellite accuracy and inconsistant survey effort we present only data from January 2010 to July 2018 on four groups. Group Composition is presented in Table 1. Gibbons were identfied using photographs, presence of infants and distinguishing features to create ID sheets for each gibbon in each group including data on HR location and singing trees. ID Book is provided in S1 Table. Only adult gibbons were selected as the focal animal.

**Table 1:** Group composition of the four main groups over the duration of the study. AdF = adult female, AdM—adult male, S = subadult, J = juvenile, I = dependent infant.

	A	C	K	M
**2010**	AdF, AdM, SM, JF	AdF, AdM, SF, JM	AdF, AdM, SF, JF	AdF, AdM, SF, JM, IM
**2011**	AdF, AdM, SM, JF	AdF, AdM, SF, JM	AdF, AdM, SF, JF	AdF, AdM, SF, JM, IM
**2012**	AdF, AdM, SM, JF, IF	AdF, AdM, SF, JM, IM	AdF, AdM, SF, JF, IF	AdF, AdM, SM, JM,
**2013**	AdF, AdM, SM, JF, IF	AdF, AdM, SF, JM, IM	AdF, AdM, SF, JF, IF	AdF, AdM, SM, JM,
**2014**	AdF, AdM, SF, JM,	AdF, AdM, SM, JM,	AdF, AdM, SF, JF	AdF, AdM, SM, JM,
**2015**	AdF, AdM, SF, JM,	AdF, AdM, SM, JM,	AdF, AdM, SF, JF	AdF, AdM, SM, JF, IF
**2016**	AdF, AdM, SF, JM,	AdF, AdM, SM, JM	AdF, AdM, SF, IF, IF	AdF, AdM, SM, JF, IF
**2017**	AdF, AdM, SM, IM	AdF, AdM, SM, JM, IM	AdF, AdM, SF, IF, IF	AdF, AdM, SF, JF
**2018**	AdF, AdM, SM, IM	AdF, AdM, SM, JM, IM	AdF, AdM, SF, IF, IF	AdF, AdM, SF, JF

Two groups (Group C and Group K) were followed extensively, yielding a nine-year dataset for inter-year comparison of home- and core-range fluctuations; two other groups yielded enough home-range data points to conduct home-range analysis (Table 2). Gibbon groups were located before dawn using their calls as a reference if no sleeping tree location (i.e. the last known location of the group in which they slept from the previous day’s follow). Gibbons are followed by two researchers who share data collection with one researcher focussing on the focal gibbon and the second researcher collecting GPS data. Instantaenous focal activity data [60] were collected every 5 minutes on a focal adult chosen via an alternate selection method (i.e., the focal adult chosen to be followed on day x_1_ would be chosen again on day x_3_, etc.) and positional data were collected using hand-held Global Positioning System units (Garmin GPS 12XL, and 60Csx with an accuracy of minimum 5–8 meters at each locality) during daily follows, on the same 5 minute instant, and also at every feeding tree (see [61]). Signal is sufficient to obtain accurate GPS locations at 5-min intervals. If the accuracy was >8m then the point was discarded. Hand-drawn maps are also used on every behavioural follow of gibbons. By splitting the data collection and having 2 researchers we are able to obtain accurate positional and behavioural data on the habituated groups [61]. All GPS locality data were converted into ‘xy’ coordinates using DNR GPS Application (University of Minnesota 2012). Estimates of home-range sizes were obtained using the kernel density estimate method [62] using the ‘adeHabitatHR’ package [63] in the statistical software package ‘R’ [64]. A volume contour of 95% of kernels analysed was considered the home range a volume contour of 50% of kernels analysed was considered the core range. The home-range analysis used the ‘href’ bandwidth for kernel smoothing. For further details see [63].

**Table 2:** Summary data of all four groups including survey effort [years and datapoints].

	# Years data	Average waypoints/year	Total # waypoints
**A**	3	109	326
**C**	9	1,946	17,517
**K**	9	1,901	17,111
**M**	1	NA	567
	**TOTAL**		**35,521**

## Results

### HR overlap

Overlap ranged from 3.52% to 23.97% of the total home range (Table 3). Home Range values for each group for each study year and change in size of HR are available in S2 Table (all in Km^2^).

**Table 3:** Overlap between study groups.

	**C**	**K**	**M**
**A**	3.52 ha (4.3%)	13.69 ha (16.8.3%)	
**C**	0	14.08 ha (23.97%)	
**K**		0	19.82 ha (20.93%)
**M**			0

### Home range size and changes over time

**Fig 2:**
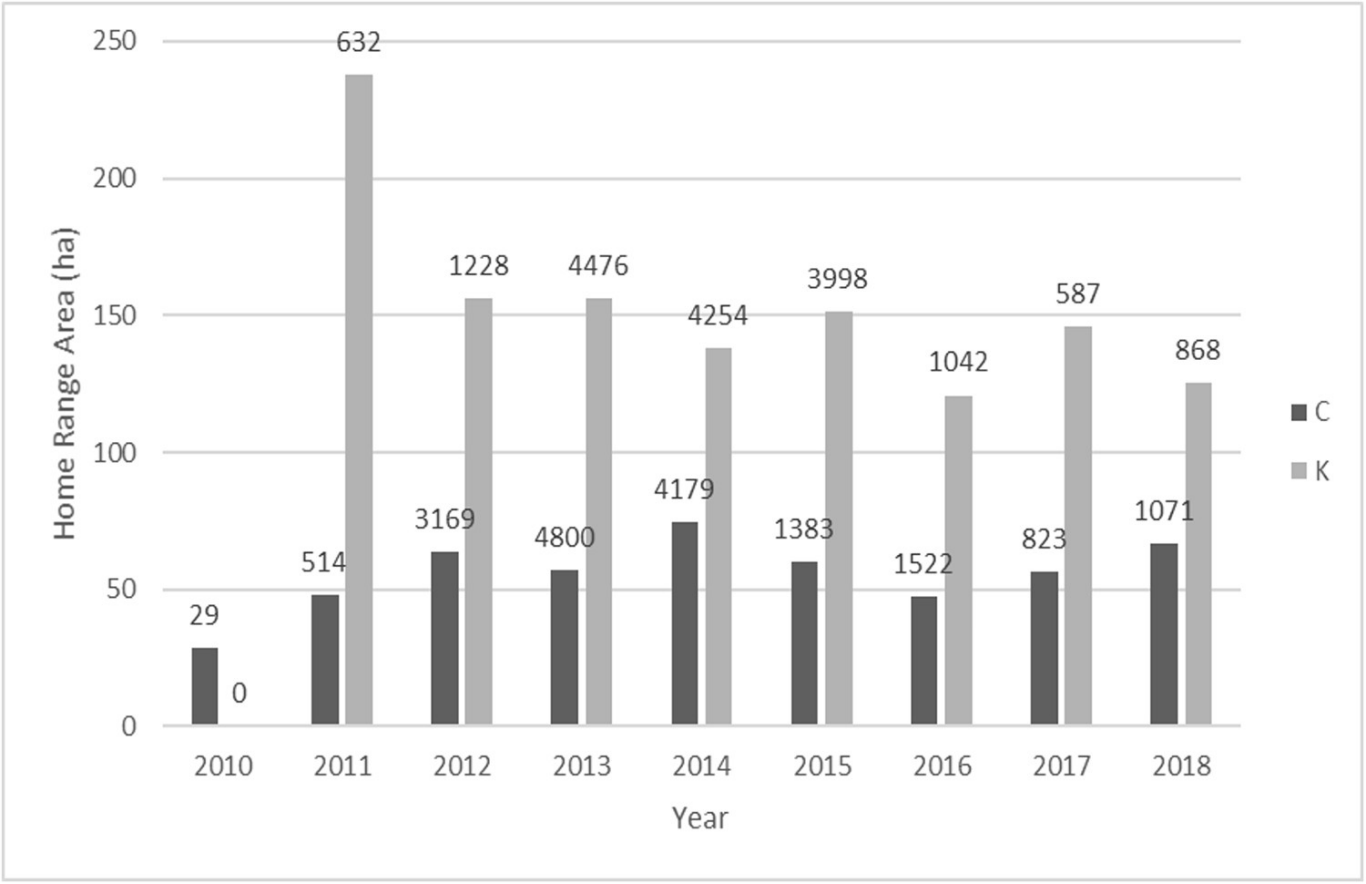
Average 95% HR size for all years and number of GPS points.

**Fig 3:**
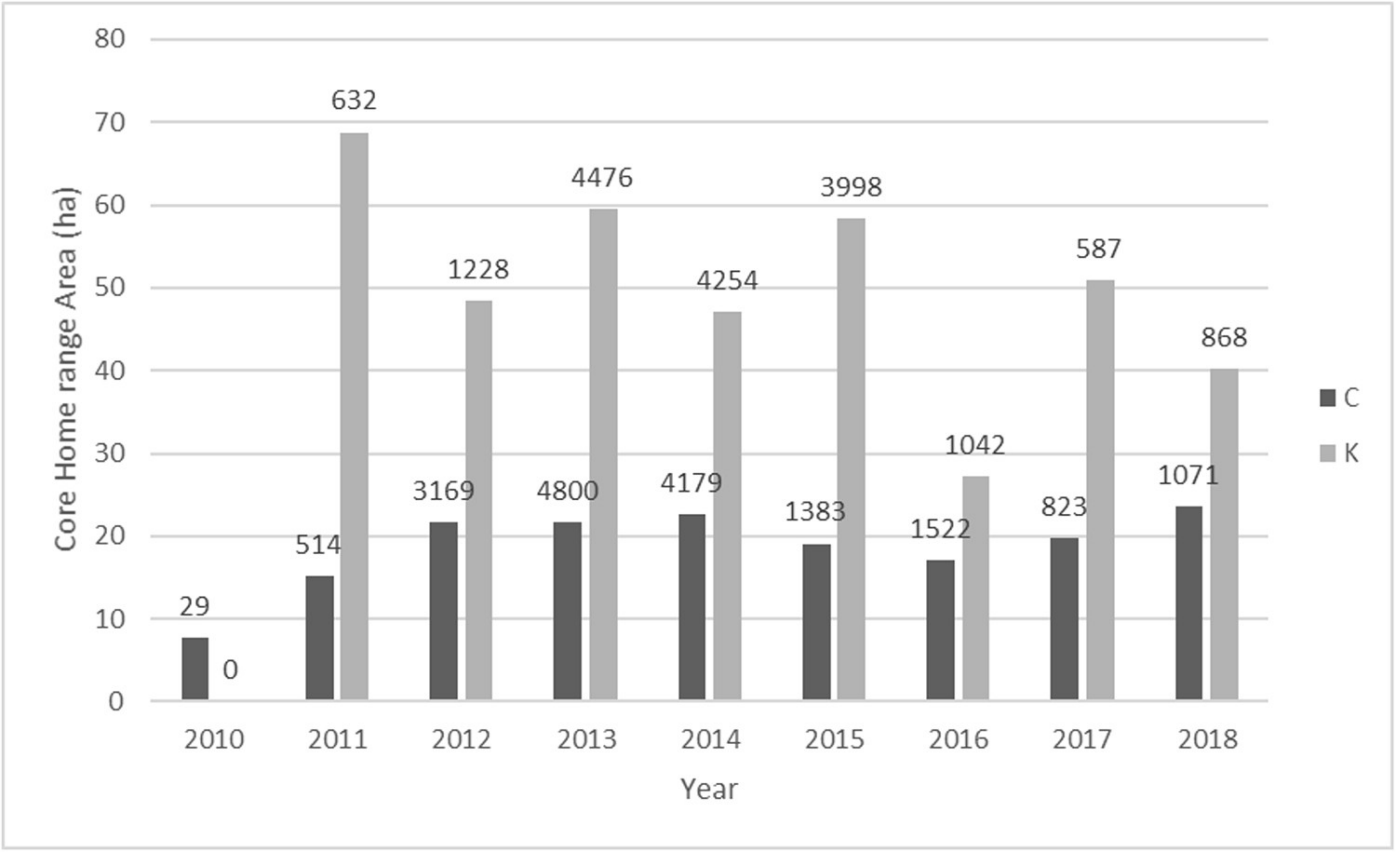
Average 95% core size for all years and number of GPS points.

Home-range and core-range sizes for Group C and Group K were relatively stable over time (Figs 2 and 3): Group C Core 18.89ha (range 7.68–23.58ha) HR 55.84ha (range 28.66–74.74ha) and Group K Core 50.07ha (range 27.24–68.79ha) HR 153.96ha (range 120.75–237.73ha).

**Fig 4:**
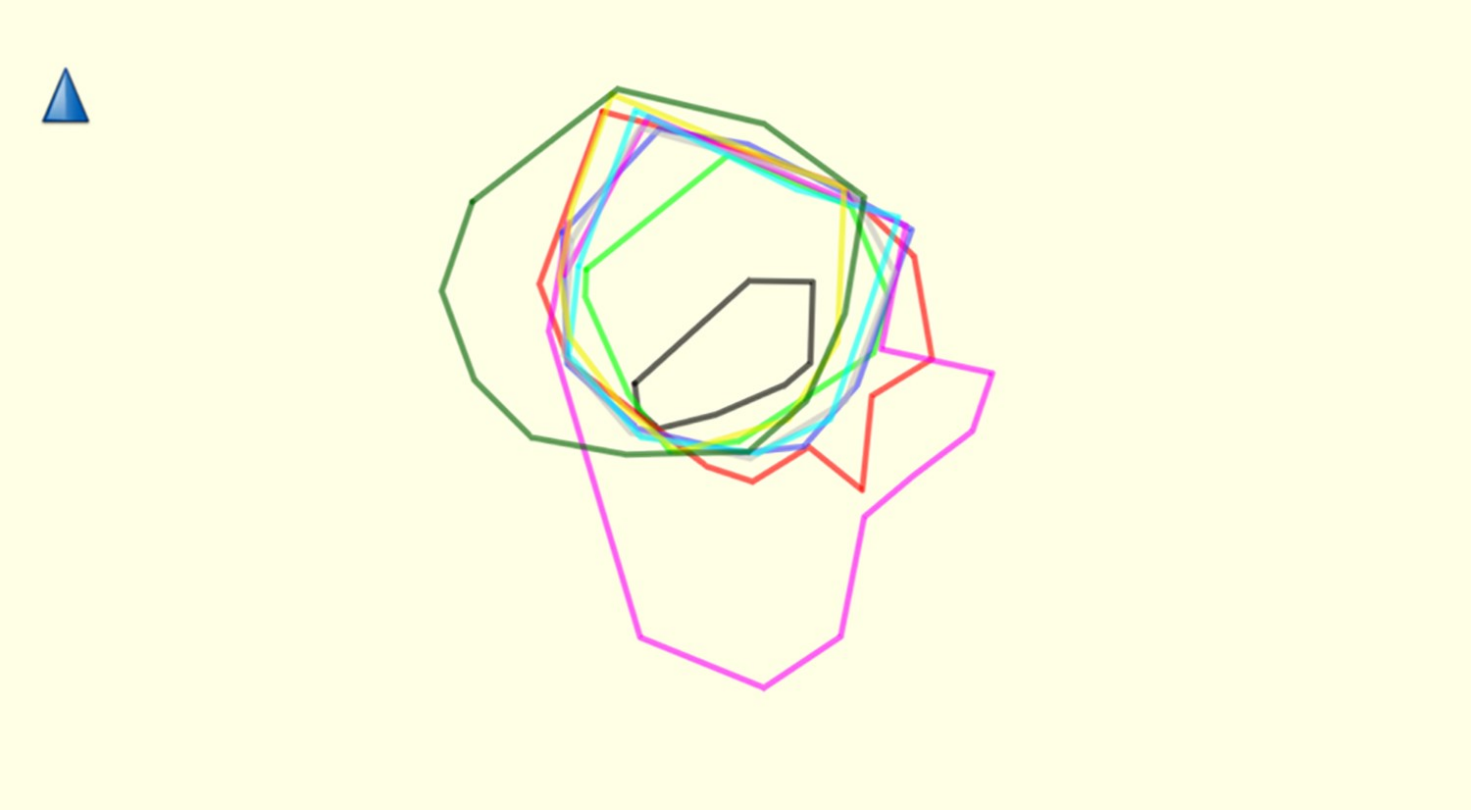
Showing site fidelity of Group C. 2010 = black, 2011 = light green, 2012 = red, 2013 = blue, 2014 = pink, 2015 = light grey, 2016 = light blue, 2017 = yellow and 2018 = dark green. Created using Garmin BaseCamp V 4.7.0.

The variation in GPS localities year on year is unlikely to cause error in home-range estimation. Core-ranges localities were fairly constant (Fig 4).

**Fig 5:**
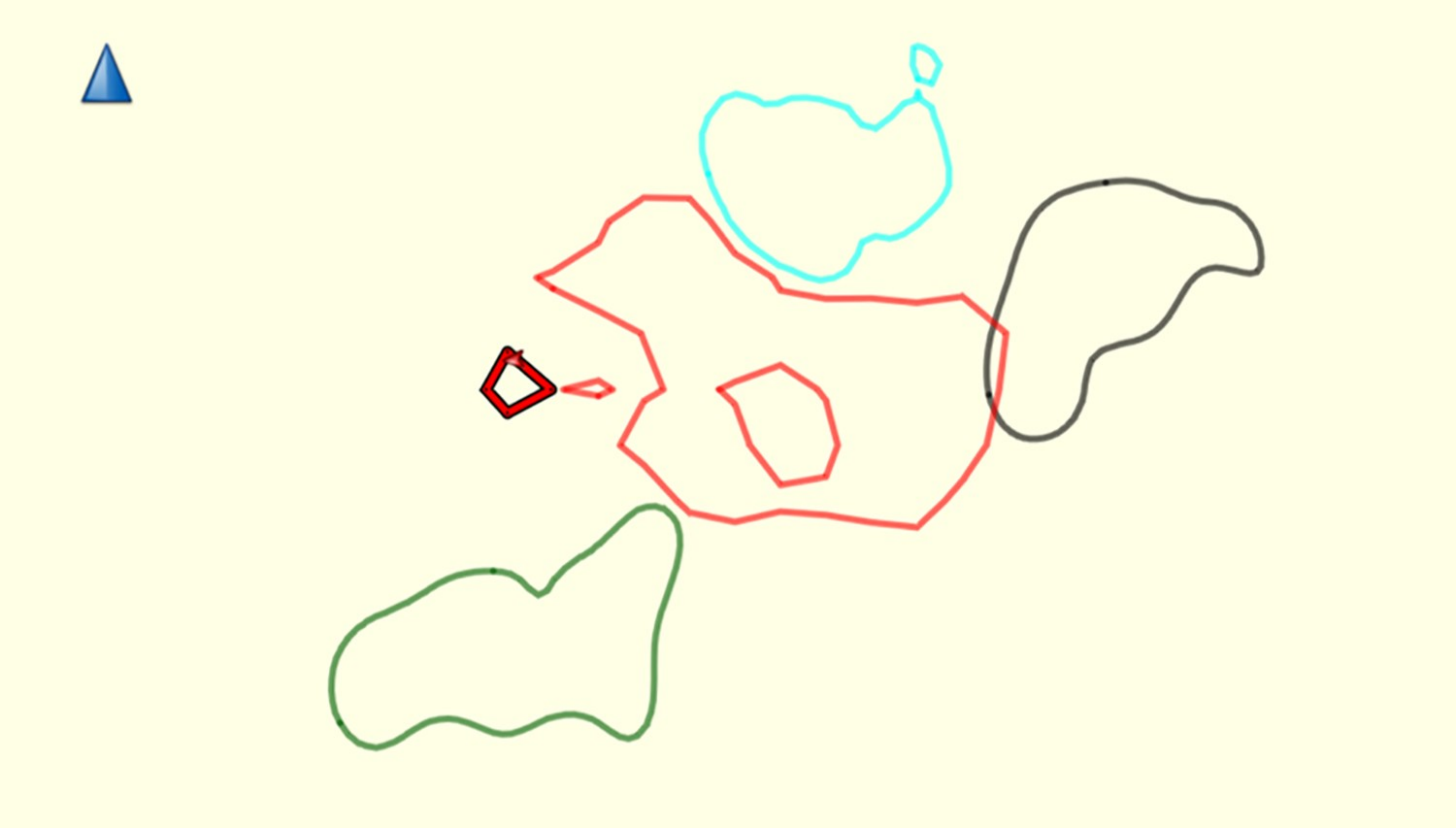
Average 50% core HR size for all years and number of GPS points. Group A = Black; Group C = Light Blue; Group K = Red; Group M = Green. Created using Garmin BaseCamp V 4.7.0.

Gibbons had consistant site fidelity for their home- and core ranges; percentage overlap ranged from 4.3 to 23.97% with a mean 16.5 ± SD 8.65% overlap in home-range area (Fig 5).

**Fig 6:**
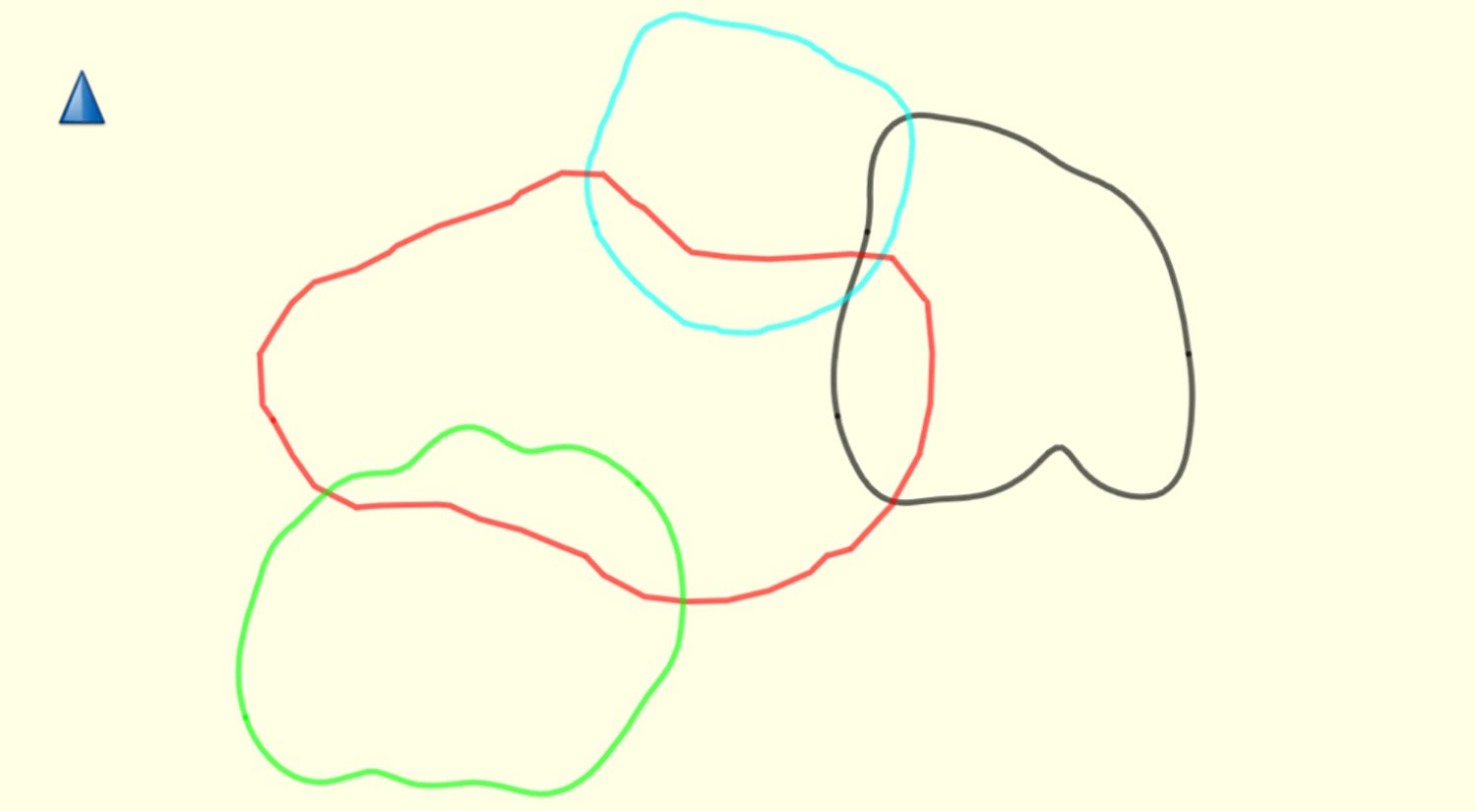
Average 95% HR size for all years and number of GPS points. Group A = black, Group C = light blue, Group K = red and Group M = green. Created using Garmin BaseCamp V 4.7.0.

Core ranges did overlap: Group K and C, in which a 0.14ha overlap (equal to 23.72% of Group C HR and 9.45% of Group K HR) occurred and Group K and M where there was a 0.2ha overlap (equal to 13.51% of Group K HR and 21.05% of Group M HR). The total forest area covered by each group remains stable over the course of the study, as does overlap (Fig 6).

### HR and fire

**Fig 7:**
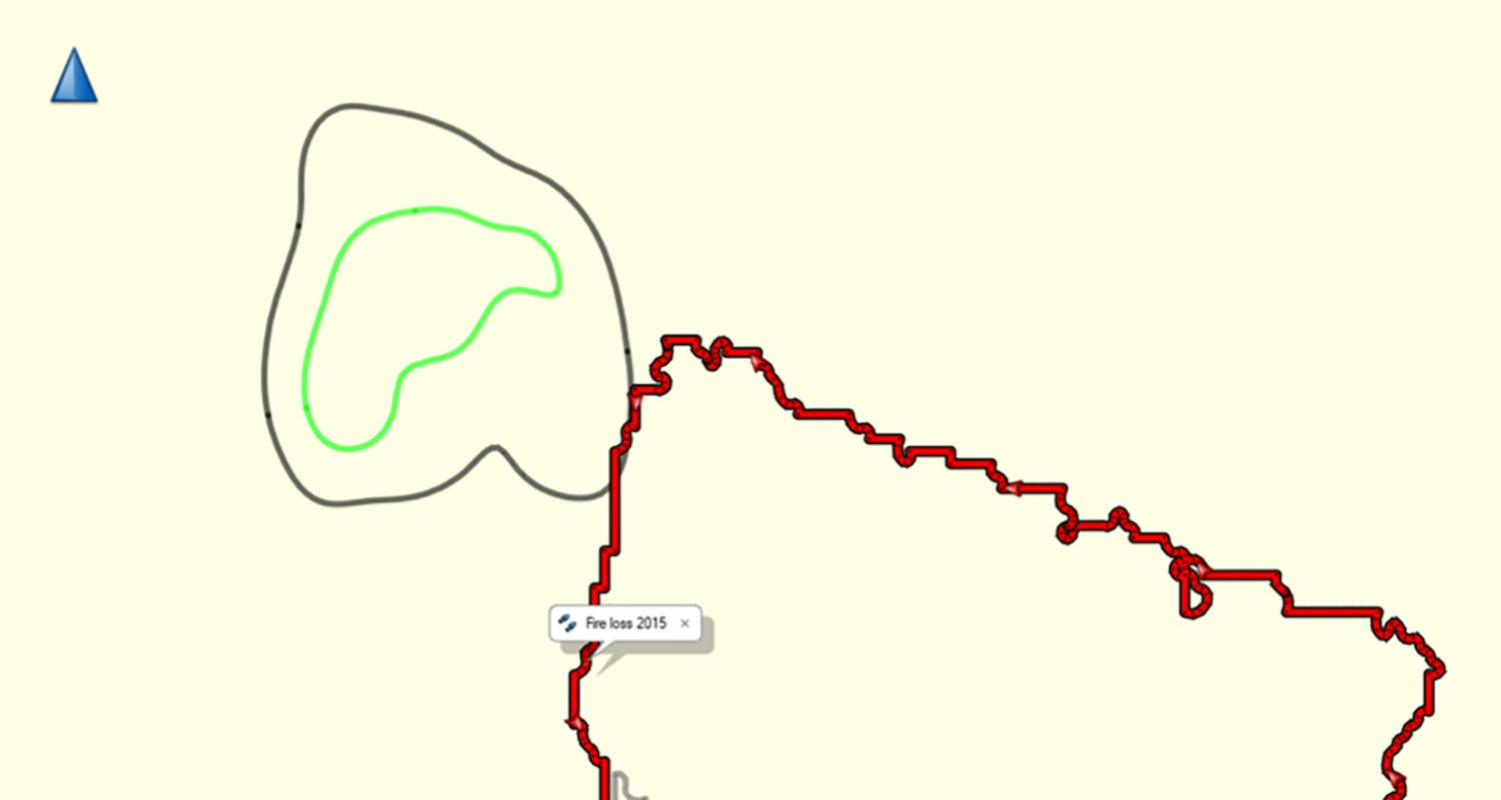
Forest loss to fire (red area) and HR of Group A (50% green, 95% black). Created using Garmin BaseCamp V 4.7.0.

In the fires in 2015 there was a loss of 10% of the forest (53.8km^2^, BNF unpublished data). For the groups for which there are data pre-and post 2015 fires was virtually no change in the HR location of Groups C or K following forest loss in the 2015 fires, but there was a change in HR of Group A as the fires directly affected the HR of Group A (Fig 7). Group A appear to have moved west away from the burnt area.

## Discussion

The HR of the gibbons from Sebangau are the largest of all comparable *Hylobates* sp. studies (Table 4). A possible explanation is the peat-swamp habitat and associated variable food availability [18,56,65–68] and/or the population density of the area being lower than carrying capacity due to anthropogenic disturbances e.g. logging and fire [43,69–71].

**Table 4:** Comparison of gibbon Hr sizes across species. Studies were selected which use similar methods for HR estimation.

Species	Country	Site and # groups included	Average HR km^2^	Reference
***Hoolock hoolock***	India	Garo Hills (7)	0.003	[72]
***Hylobates lar***	Indonesia	Khao Yai (11)	0.180	[73]
***Hylobates lar***	Thailand	Khao Yai (2)	0.230	[74]
***Hylobates agilis***	Indonesia	Limau Manis (2)	0.240	[75]
***Hylobates albibarbis***	Indonesia	Gunung Palung (3)	0.280	[76]
***Hylobates lar***	Thailand	Khao Yai (7)]	0.280	[77]
***Hylobates moloch***	Indonesia	Gunung Halimun-Salak National Park (3]]	0.370	[78]
***Hylobates albibarbis***	Indonesia	Sebangau (7]	0.96	This study
***Nomascus nasutus***	China	Cao Vit Gibbon Conservation Area [Vietnam] and Bangliang Gibbon Nature Reserve China (6]	1.300	[79]
***Nomascus concolor***	China	Mt. Wuliang (1]	1.500	[80]
***Nomascus hainanus***	China	Hainan (3]	4.850	[81]
***Nomascus leucogenys***	China	Yunnan (2]	5.400	[82]

Core areas were where the gibbons slept and sang their morning duets. These areas were vigourously defended and do not overlap [61,83] whereas the home range area did overlap with other groups and feeding trees were shared between groups [trees are tagged with unique numbers]. Thus feeding takes place across both the core and HR of the territory, singing and sleeping only in the core and all is defended. None of the intergroup encounters occur in the core of any of the four groups [18,30,46].

We demonstrate that gibbons have significant site fidelity for their home ranges, they do have consistent overlap with neighbouring groups [61,77] and there is some annual movement for all groups in the position of the centrepoint of their HR. Unsurprisingly forest loss from fire does affect the location of the HR of the impacted group, but does not appear to affect adjacent groups, though more data are needed on this. Given how fixed HR’s are for gibbons, loss of forest will affect the size of the HR, the ability for the group to access food and ultimatley could result in groups being compressed into a small area of suitable forest surrounded by unsuitable forest, creating over-crowding and limited dispersal options for sub-adults [84]. Hence why the average HR overlap of 18.76% is much smaller than that reported from other studies: 65% [77] and 79% [75].

The fires did not impact the territories of Groups C, K or M due to fires being brought under control by teams of fire-fighters. Despite this effort, some of the forest did burn in the area of Group A’s territory, thus pushing the group further west. There are major negative impacts of Borneo peat/forest fire on biodiversity, affecting large numbers of species from Kalimantan-wide to site scale. Known/suspected impacts include respiratory ailments in animals, e.g. orangutans [Borneo Orangutan Survival Foundation, pers. comm.]; reduced gibbon territorial singing [85] and orangutan calling [86] and changes in phenology [87,88]. These and other as yet unknown impacts are expected to continue and worsen unless the underlying causes can be tackled, adding an additional major threat to biodiversity and increasing extinction risk for many species. Understanding the complex use of space of these territorial animals is important in assessing both carrying capacity [43,89], how dispersing gibbons use space and establish a territory [9,90–92] and understading how reintroduced gibbon pairs will establish their core and HR [93–97].

Hunting, fire, forest clearance and forest fragmentation are all impacting Borneo’s gibbons. Gibbons need large areas to survive and linking forests and reducing fragmentation is the key to their conservation. Landscapes and connectivity depend on collaboration between local and international governments, communities, conservation organizations and researchers. As we understand more about gibbon habitat use we will begin to see how best to connect the remaining forest.

## Supporting information

S1 Table Identification tables for Group C as an example of how gibbons are distinguished from each other. (DOCX)

S2 Table Home Range values for each group for each study year and change in size of HR (all in Km^2^). (DOCX)
