# Supplementary figures and images for: Home range variation and site fidelity of Bornean southern gibbons [Hylobates albibarbis] from 2010-2018
Source: PLoS One. 2019 Jul 31;14(7):e0217784. doi: 10.1371/journal.pone.0217784 (PMC6668788; doi:10.1371/journal.pone.0217784)

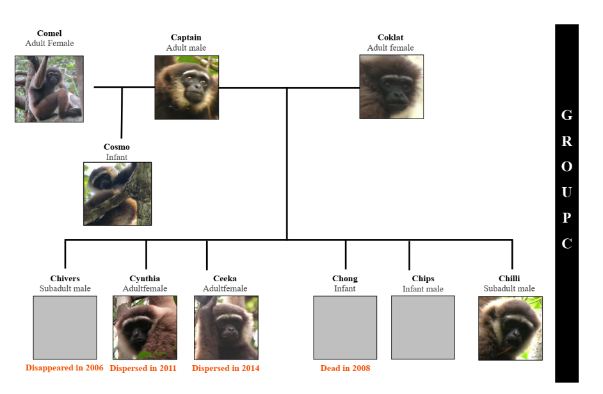


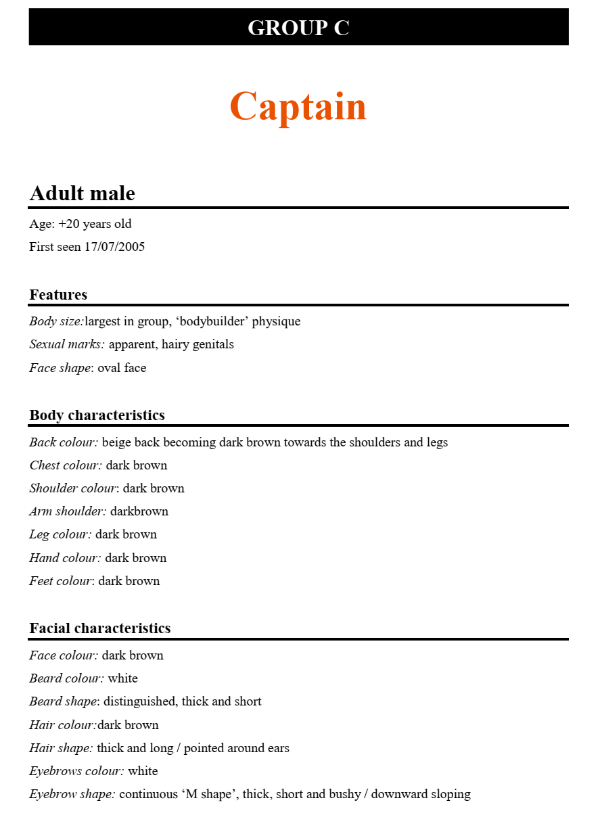

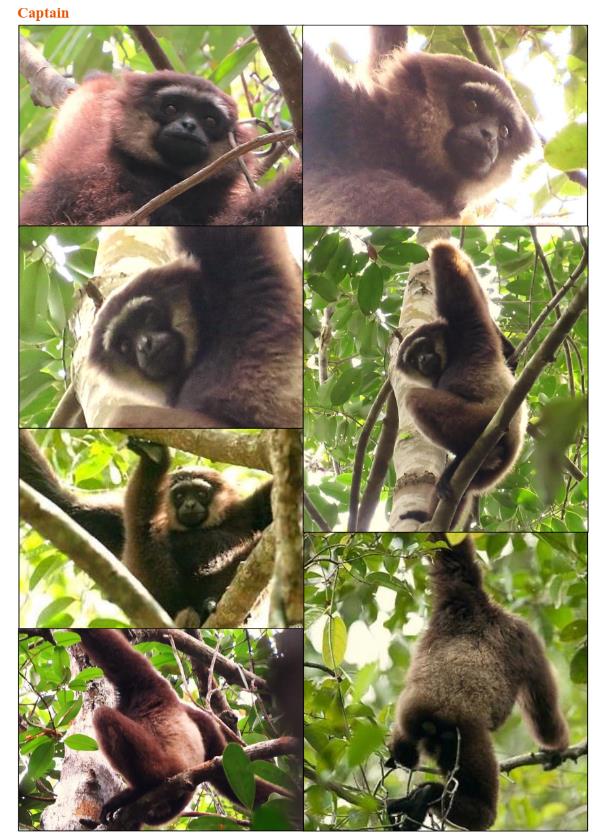

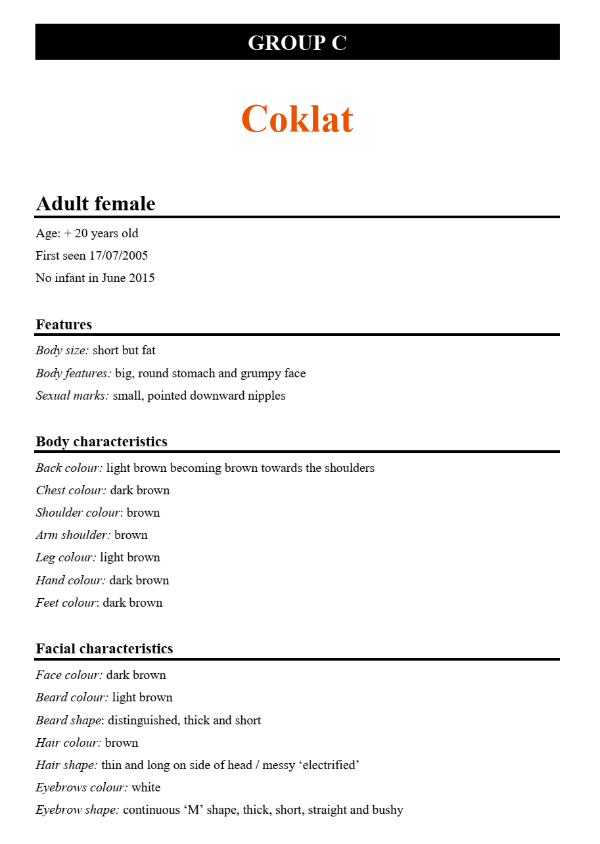

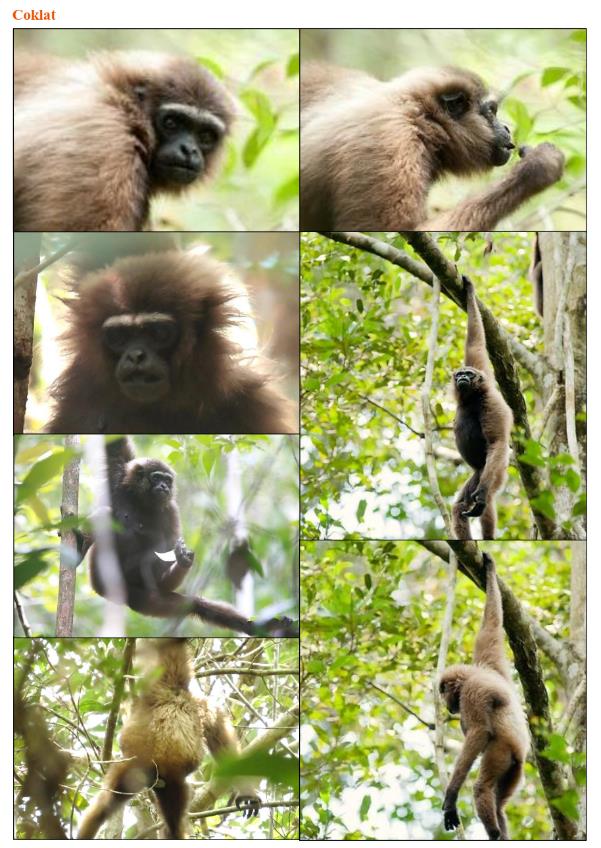

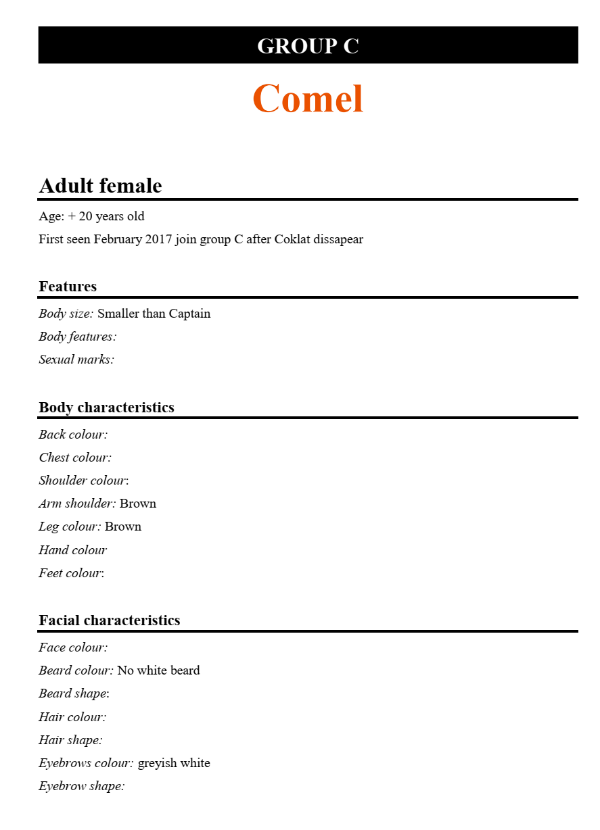

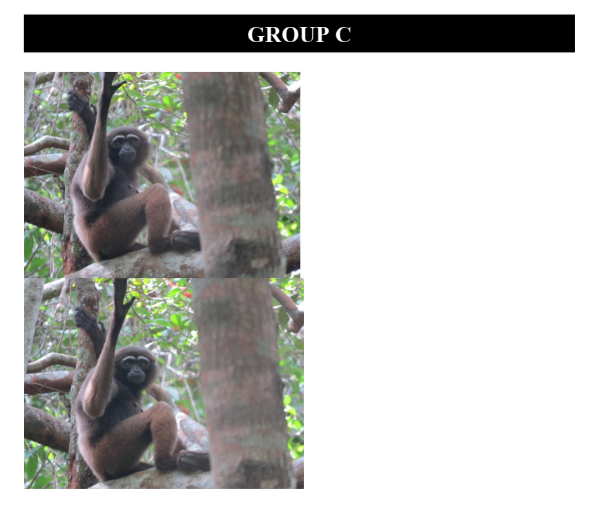

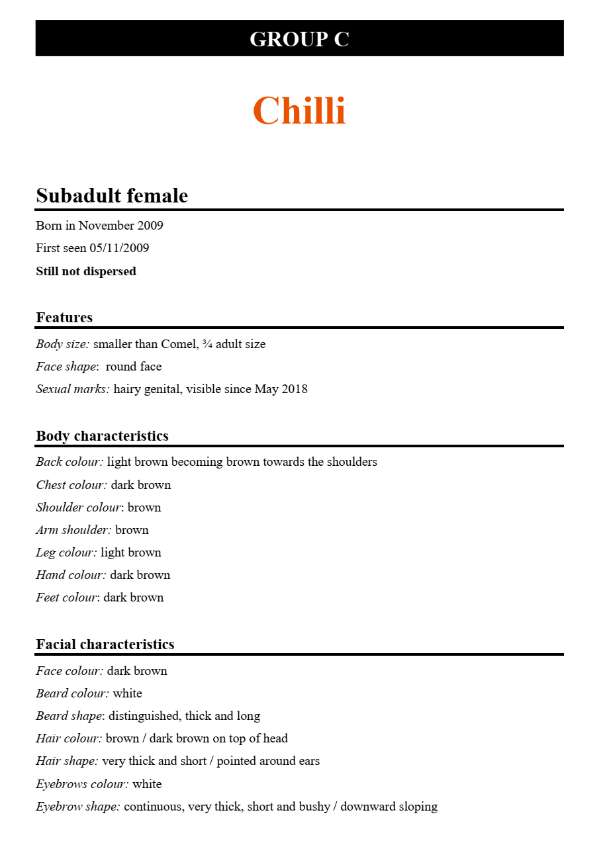

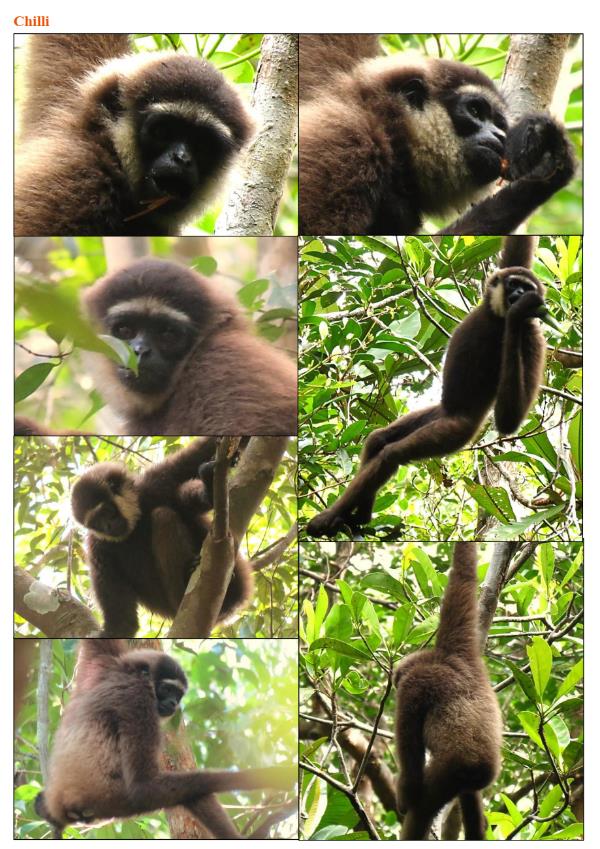

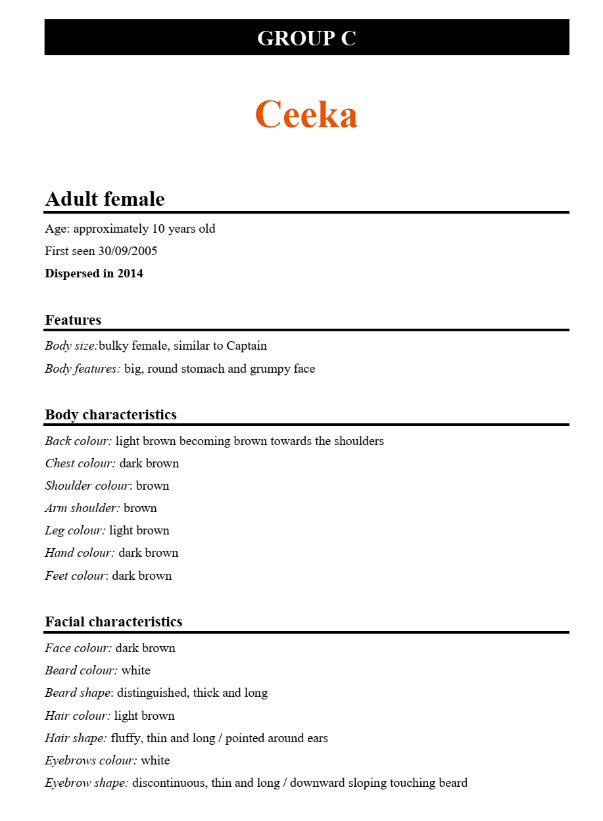

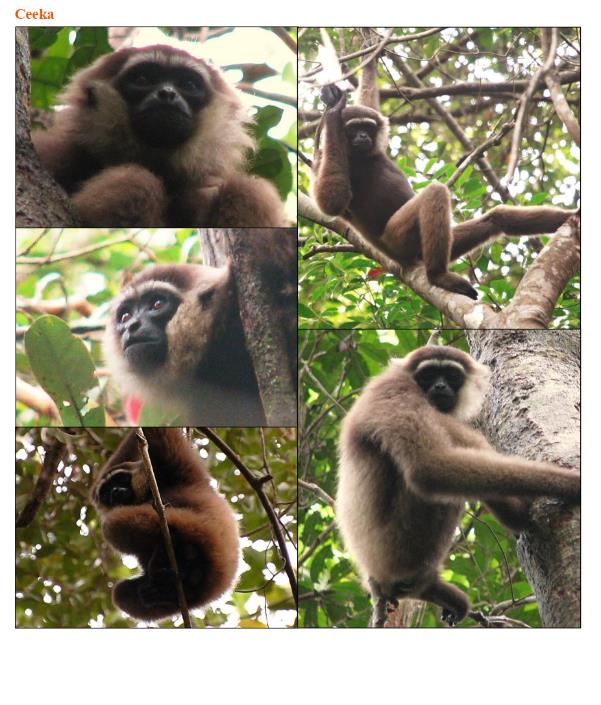

Supplement: S1 Table — (DOCX) [file pone.0217784.s001.docx]
