# Supplementary material for: Home range variation and site fidelity of Bornean southern gibbons [Hylobates albibarbis] from 2010-2018
Source: PLoS One. 2019 Jul 31;14(7):e0217784. doi: 10.1371/journal.pone.0217784 (PMC6668788; doi:10.1371/journal.pone.0217784)
Supplement: S2 Table — (DOCX) [file pone.0217784.s002.docx]

**Supplement Table S2**

Home Range values for each group for each study year and change in size of HR [all in Km^2^].

|  | **2010** | | **2011** | | **2012** | | **2013** | | **2014** | | **2015** | | **2016** | | **2017** | | **2018** | | |
| --- | --- | --- | --- | --- | --- | --- | --- | --- | --- | --- | --- | --- | --- | --- | --- | --- | --- | --- | --- |
| **Group** | **Km^2^** | **Change in HR size** | **Km^2^** | **Change in HR size** | **Km^2^** | **Change in HR size** | **Km^2^** | **Change in HR size** | **Km^2^** | **Change in HR size** | **Km^2^** | **Change in HR size** | **Km^2^** | **Change in HR size** | **Km^2^** | **Change in HR size** | | **Km^2^** | **Change in HR size** |
| **A** | 0.0098 | NA | 0.02 | 0.0102 | NA | NA | NA | NA | 0.37 | 0.35 | NA | NA | NA | NA | 1.35 | 0.98 | | 0.4 | -0.95 |
| **C** | 0.29 | NA | 0.74 | 0.45 | 1 | 0.26 | 1.02 | 0.02 | 1.56 | 0.54 | 0.77 | -0.79 | 0.74 | -0.03 | 0.77 | 0.03 | | 0.97 | 0.2 |
| **E** | 0.3 | NA | NA | NA | NA | NA | NA | NA | NA | NA | NA | NA | NA | NA | NA | NA | | NA | NA |
| **J** | NA | NA | 0.21 | NA | 0.47 | 0.26 | NA | NA | NA | NA | NA | NA | NA | NA | NA | NA | | NA | NA |
| **K** | 0.31 | NA | 1.81 | 1.5 | 1.49 | -0.32 | 1.68 | 0.19 | 1.82 | 0.14 | 1.64 | -0.18 | 1.37 | -0.27 | 1.46 | 0.09 | | 1.55 | 0.09 |
| **M** | NA | NA | NA | NA | 1.04 | NA | NA | NA | 0.14 | -0.9 | NA | NA | NA | NA | NA | NA | | NA | NA |
| **T** | NA | NA | 0.19 | NA | NA | NA | NA | NA | NA | NA | NA | NA | NA | NA | NA | NA | | NA | NA |

Appendix 2 HR centre point changes over time [5 groups]. D = distance in km^2^, B = bearing in degrees.

| **GROUP** | **2010** | | **2011** | | **2012** | | **2013** | | **2014** | | **2015** | | **2016** | | **2017** | | **2018** | |
| --- | --- | --- | --- | --- | --- | --- | --- | --- | --- | --- | --- | --- | --- | --- | --- | --- | --- | --- |
|  | D | B | D | B | D | B | D | B | D | B | D | B | D | B | D | B | D | B |
| **A** | First HR data | | 0.43 | 40 | NA | | NA | | 0.27 | 220 | NA | | NA | | 0.17 | 180 | 0.33 | 350 |
| **C** | First HR data | | 0.12 | 15 | 0.01 | 85 | 0.04 | 290 | 0.32 | 167 | 0.33 | 348 | 0.01 | 328 | 0.07 | 301 | 0.16 | 265 |
| **J** | NA | | First HR data | | 0.64 | 286 | NA | | NA | | NA | | NA | | NA | | NA | |
| **K** | First HR data | | 0.06 | 100 | 0.41 | 274 | 0.27 | 41 | 0.08 | 269 | 0.38 | 55 | 0.63 | 145 | 0.82 | 294 | 0.18 | 126 |
| **M** | NA | | NA | | First HR data | | NA | | 0.33 | 348 | NA | | NA | | NA | | NA | |
